# MATH: A Deep Learning Approach in QSAR for Estrogen Receptor Alpha Inhibitors

**DOI:** 10.3390/molecules28155843

**Published:** 2023-08-03

**Authors:** Rizki Triyani Pusparini, Adila Alfa Krisnadhi, Firdayani

**Affiliations:** 1 Tokopedia-UI AI Center of Excellence, Faculty of Computer Science, Universitas Indonesia, Depok 16424, Indonesia; 2 Research Center for Vaccine and Drugs, Research Organization for Health, National Research and Innovation Agency (BRIN), Jakarta 10340, Indonesia; firdayani@brin.go.id

**Keywords:** artificial intelligence, molecular graph structure, Transformer, estrogen receptor alpha, breast cancer, QSAR

## Abstract

Breast cancer ranks as the second leading cause of death among women, but early screening and self-awareness can help prevent it. Hormone therapy drugs that target estrogen levels offer potential treatments. However, conventional drug discovery entails extensive, costly processes. This study presents a framework for analyzing the quantitative structure–activity relationship (QSAR) of estrogen receptor alpha inhibitors. Our approach utilizes supervised learning, integrating self-attention Transformer and molecular graph information, to predict estrogen receptor alpha inhibitors. We established five classification models for predicting these inhibitors in breast cancer. Among these models, our proposed MATH model achieved remarkable precision, recall, F1 score, and specificity, with values of 0.952, 0.972, 0.960, and 0.922, respectively, alongside an ROC AUC of 0.977. MATH exhibited robust performance, suggesting its potential to assist pharmaceutical and health researchers in identifying candidate compounds for estrogen alpha inhibitors and guiding drug discovery pathways.

## 1. Introduction

The National Cancer Institute estimates for the United States for 2023 are that approximately 297,790 new cases of invasive breast cancer will be diagnosed in women and that 43,170 women will die from breast cancer [1]. From 2017 to 2022, 7.8 million living women were diagnosed with breast cancer, making it the most prevalent cancer, globally [2].

Approximately 80% of breast cancer cases are estrogen-receptor-(ER)-positive [3]. In those cases, the proliferation of cancer cells is stimulated by estrogen receptor alpha (ERα), a protein activated by the estrogen hormone. Consequently, endocrine therapy is often employed as one of the treatment choices. Prescribing an appropriate endocrine therapy requires finding the necessary active compounds, called ERα inhibitors, that can block the growth-increasing effect of the estrogen hormone on breast cancer cells: in effect, this can slow or even stop the cancer progression completely. This treatment approach may be preferred over chemotherapy, because it often uses less toxic drugs than those used in chemotherapy [4].

However, finding the appropriate ERα inhibitors is generally challenging and time-consuming, because of the large amounts of in vitro trial-and-error needed. Modern drug design shortens the time needed to find the appropriate key compounds, by employing various computer-aided analyses before in vitro. One such analysis is called QSAR, whereby one can predict the desired response variables (Y), such as physicochemical properties, bioactivity, toxicity, and chemical reactivity [5,6,7,8,9], based on a set of molecular descriptor properties as the predictor variables (X) [10].

Nowadays, many QSAR developments apply a multi-objective QSAR approach to drug discovery [11]. Traditional QSAR methods have transitioned towards machine learning (ML) models, including deep learning (DL) models, to achieve more diverse variations in the resulting predictors. ML, which encompasses DL as a subset, allows the construction of models directly from the data, without assuming specific data distributions. These models use large datasets and complex algorithms to identify patterns and relationships between chemical structures and biological activity [12].

The State-of-the-Art DL method for QSAR analysis of ERα inhibitors is the so-called molecule-attention Transformer (MAT) proposed by Maziarka et al. [13], which is based on the Transformer model [14]. The original Transformer was originally intended to model sequence-to-sequence problems, by predicting an output sequence based on some input sequence [15]. The key aspect of this model is using self-attention scoring among all the sequence elements, which enables the model to understand contextual relationships between them. This capability allows the Transformer to be used extensively in natural language processing, such as machine translation, sentiment analysis, etc. Motivated by the use of SMILES, which encodes chemical molecules as sequences, MAT adapted the Transformer to the problem of predicting ERα inhibitors from a given SMILES encoding of chemical compounds, by adding self-attention scoring based on inter-atomic distances and molecular graph structures.

This paper proposes MATH (molecule-attention Transformer plus hydrogen bond), an improvement of MAT, for predicting ERα inhibitors by augmenting self-attention scoring with intramolecular hydrogen bond information. This modification stems from the observation by Kuhn et al. [16] that intramolecular hydrogen bonds (H-bonds) also strongly influence the interaction between chemical compounds. A particular H-bond’s strength depends on the donor and acceptor species, the environment, and the interaction angle. H-bonds are important in drug receptor interactions and in the structural integrity of many biological molecules [17].

We compared MATH to three baseline models for classifying estrogen receptor alpha inhibitors. The first baseline was MAT, by Maziarka et al. [13], which does not take into account the strength of intramolecular hydrogen bonds in the candidate compounds. In addition, we also performed a comparison to the SMILES Transformer work, by Honda et al. [18], who trained a Transformer by decoding textual representations, in an attempt to reproduce the results from Maziarka et al. [13] Finally, similarly to what Honda et al. [18] had done in their study, we also trained, as the third baseline, an MLP model whose input was the ECFP of the candidate compounds. The fingerprint itself was developed by Rogers and Hahn [19], and it is often assumed to contain the strongest predictors for molecular property prediction problems.

Our research contributes to the classification of candidate compounds as estrogen receptor alpha inhibitors in breast cancer therapy, by introducing intramolecular hydrogen bond information, using two representational approaches:

- The first approach incorporates hydrogen bond presence as a Boolean matrix, providing insights into the compound’s structure and interaction with the estrogen receptor.
- The second approach includes detailed intramolecular hydrogen bond information, quantifying bond strength through donors, acceptors, and distances.

By integrating intramolecular hydrogen bond information, our model’s accuracy is an improvement, facilitating drug research, discovery pathways, and identification of active compounds for breast cancer therapy.

The initial section of our study investigates related work, serving as the foundation for method selection. The second section elucidates the intricacies of data collection and preprocessing techniques, while comprehensively outlining the two approaches employed in MATH: one utilizing Boolean representation, and the other incorporating various threshold variations, with each approach being assessed alongside corresponding evaluation metrics. Next, we compare MATH performance against the baseline method. Finally, our findings are discussed, and potential future directions explored.

## 2. Related Work

In recent years, various approaches have been proposed for classifying and predicting the bioactivity of compounds inhibiting estrogen receptor alpha, ranging from the QSAR modeling approach to artificial intelligence (AI) methods, such as machine learning (ML) and deep learning (DL). For example, Tong et al. [20] developed a QSAR model using CoMFA analysis to predict the binding affinity of estrogenic chemicals to ER alpha and ER beta receptors. Then, Ribay et al. [21] developed a computational model, combining the QSAR approach and the similarity search, to predict the binding potential of small molecules to estrogen receptors. Meanwhile, Cotterill et al. [22] compared the classification performance of several QSAR models, molecular docking, and molecular dynamics, in predicting the binding of endocrine-disrupting chemicals (EDCs) to estrogen receptors (ERα). Moreover, Zekri et al. [23] developed (QSAR) to investigate the relationship between indazole derivatives and estrogen receptor alpha (ERα), using multiple linear regression (MLR), and to analyze the compound structure and activity of the compound. However, the mentioned results required further experiments, to determine the predictive activity and to explore additional structural features affecting biological activity.

Research on the bioactivity of compounds has seen significant growth, using ML and DL methods. This second approach uses existing data to train predictive models. However, this approach is hindered by the lack of currently available datasets related to bio-activity [24]. Previous approaches, such as the hybrid method by Wallach et al. [25] and the domain-knowledge-based approach by Feinberg et al. [26], aimed to address this. DL has been valuable in molecular property prediction, using handcrafted representations, such as SMILES and fingerprints [27]: this technique enables virtual screening, by generating fixed-sized fingerprints of proteins and small molecules.

Furthermore, the use of deep learning in molecular property prediction is increasing. For instance, Wang et al. [28] and Honda et al. [18] have employed pre-trained Transformers [14], using text representations (SMILES) as input for molecular data. Honda et al. [18] showed that a decoding-based approach increases the efficiency of the data model. A similar method was proposed by Ciallella et al. [29], by exploring the use of fingerprints to predict compound activity. This study shows that criteria are still needed in selecting chemical descriptors. By contrast, to add information regarding the actual structure of the model and to avoid using linear (textual) molecular representations as input, we adapted MAT by Maziarka et al. [13]. They developed a Transformer with augmentation self-attention of molecular graphs to the chemical structure [14], which is essential for achieving robust empirical performance. Additionally, we employed domain-specific pre-training based on Wu et al. [30]. Table 1 provides a summary of the closest related work in the field of molecular representation methods for classifying active or inactive compounds. The three works evaluated were the SMILES Transformer (ST), the molecule-attention Transformer (MAT), and our proposed molecule-attention Transformer plus hydrogen bond (MATH). Each method aims to enhance classification accuracy by effectively representing molecular structures.

## 3. Results

We conducted two experiments, to evaluate the performance of prediction models for estrogen receptor alpha inhibitors. In the first experiment, we reproduced and compared MAT by Maziarka et al. [13], SMILES Transformer (ST) by Honda et al. [18], and extended-connectivity fingerprinting (ECFP). Additionally, we extended the MATH model, by incorporating intramolecular hydrogen bond information as parameters.

In the second experiment, we compared the performance of these four models, to assess their predictive capabilities. We aimed to determine if MATH could achieve State-of-the-Art results, making it a potential candidate for the virtual screening of estrogen-receptor compounds.

According to the first experiment, we used pre-trained weights of ST [18] and ECFP [19] for the MLP model with the ChEMBL206 dataset in CSV format. The Transformer inspires ST and learns molecular fingerprints via unsupervised pre-training of a sequence-to-sequence language model, using the vast corpus of SMILES. ECFP is commonly used because of its outstanding performance in molecular structure comparisons. Both use the SMILES fingerprints approach, which is a textual representation. We augmented the self-attention built upon MAT, to use the actual information from the structure. This allowed us to avoid the use of linearized molecules, which we expected would be a better inductive bias for the model [13]. The addition of the adjacency matrix, distance matrix, and hydrogen bond was done in MATH.

We evaluated our model’s performance in the second experiment, using precision, recall, F1 score, specificity, and ROC AUC on the test data. The specificity metric is crucial, especially for imbalanced datasets such as ours, as it helps us understand the model’s ability to identify true negatives (inactive compounds) and to distinguish them from false positives (active compounds), providing a comprehensive view of its efficacy in predicting estrogen receptor alpha inhibitors.

By incorporating specificity into our evaluation, we were able to better define the model’s applicability domain, ensuring more reliable extrapolation of predictions within the chemical space. This makes our model more robust in drug discovery and candidate screening for estrogen-receptor compounds. The results indicated that the MATH model, with a distance tolerance threshold of 3.0, outperformed the other models, regarding precision, recall, F1 score, and specificity. The comparison of the evaluation results is presented in Table 2, including results for ST, ECFP, MAT, MATH (Boolean), and MATH with performance at various thresholds (distance2.2–4.0Å).

**Figure 1:**
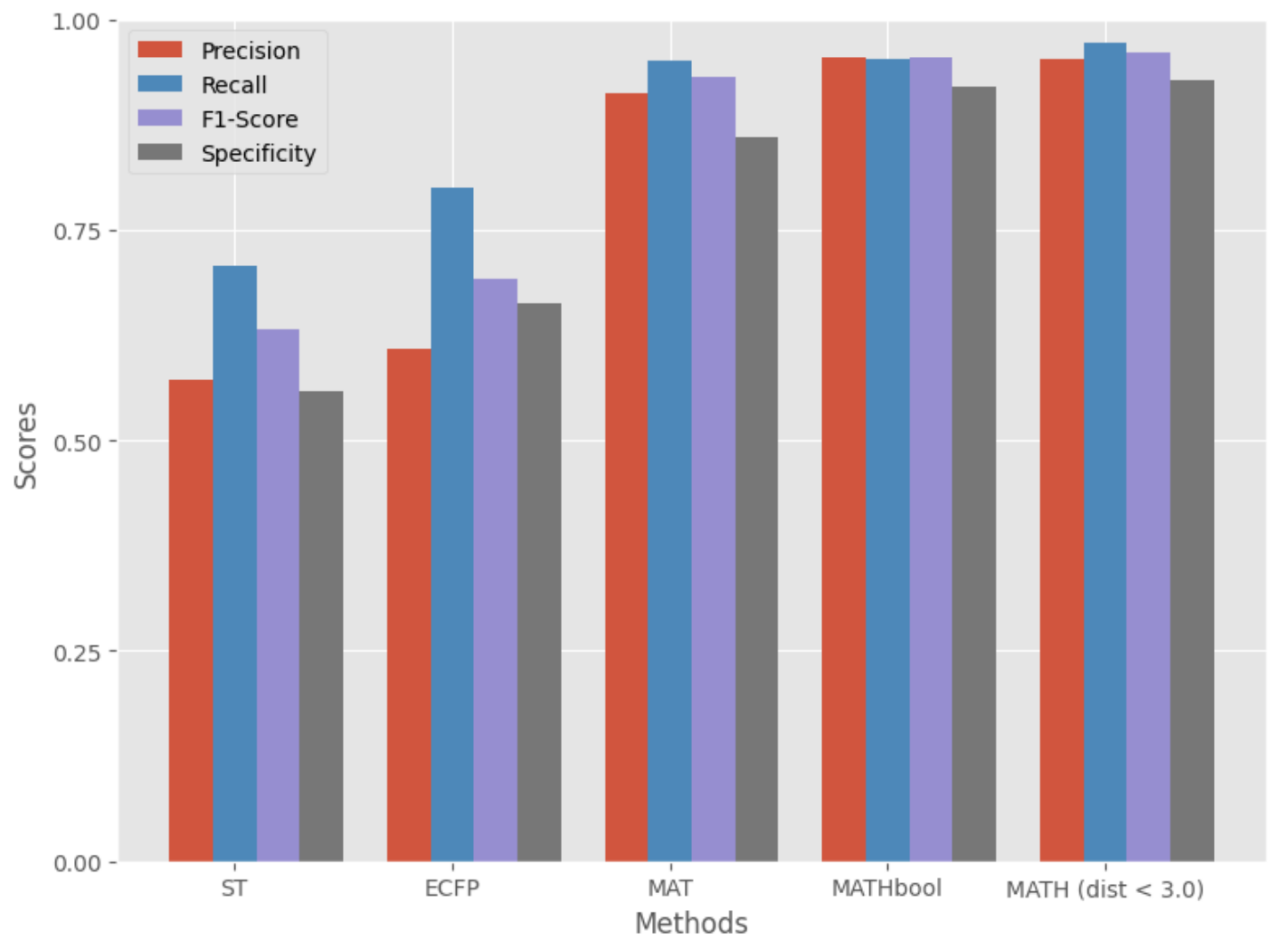
Bar chart comparing the experimental results of ST, ECFP, MAT, MATH (Boolean), and MATH (dist<3.0Å) models, highlighting the model with the highest evaluation metrics.

Figure 1 shows a bar chart of the comparison of ST, ECFP, MAT, MATH (Boolean), and MATH (dist<3.0Å), in which it can be seen that the MATH model with distance (3.0Å) outperformed the other models.

Table 3 presents the confusion matrix of MATH (dist<3.0Å) as the best-performing model, chosen based on its highest F1 score, which was achieved during five-fold cross-validation for training and testing.

## 4. Discussion

One of the therapies applied in breast cancer is endocrine therapy using estrogen receptor alpha inhibitors. Research for new drugs is ongoing, as are the limitations and weaknesses of the existing ones. The search for new compounds could be done by looking at the profiles of compounds that have been previously reported, and then examining their similarities, or by performing a QSAR evaluation. Developments in computer science have rendered it possible to apply this analysis through machine learning or deep learning approaches with much available data.

Deep learning has successfully represented molecules, using the ST, ECFP, MAT, and MATH models.

Table 2 presents evaluation metrics for models, including ROC AUC, precision, recall, F1 score, and specificity, to predict estrogen alpha inhibitors. The MATHbool model exhibited exceptional precision (0.955), recall (0.953), and F1 score (0.954). It made fewer false positive predictions, while effectively identifying the most positive instances. The MATHdist ≤3.0 model also performed impressively in precision (0.952), recall (0.972), and F1 score (0.960), achieving a good balance between identifying positive instances and minimizing false positives. For clarity, in presenting the confusion matrix, Table 3 presents the MATH (dist<3.0Å), which achieved the highest performance and best F1 score during five-fold cross-validation. This top-performing model impressively balanced precision and recall, leading to excellent performance with minimal false positives (10) and false negatives (2). Additionally, it accurately classified 181 true positive instances and 148 true negative instances. The remarkable F1 score underscores the model’s proficiency in making precise predictions, rendering it well-suited to classification tasks requiring a robust balance between precision and recall.

Specificity, crucial for true negative predictions and applicability domain, was 0.920 for MATHbool and 0.922 for MATHdist <3.0, indicating their ability to identify negative instances correctly. Compared to MAT, incorporating hydrogen bond information improved MATHbool and MATHdist <3.0 model performance across all metrics.

The ROC AUC metric assessed the models’ discriminatory power, with MATHbool and MATHdist < 3.0 performing best (0.973 and 0.977 ROC AUC, respectively), distinguishing active from inactive compounds. The ROC AUC of MATH demonstrated slight improvements, because our dataset was highly imbalanced. The imbalanced distribution of classes compounds the problem of overlap and makes classification an even more challenging task [31]. When applied to imbalanced data, ROC can depict the overly optimistic performance of classifiers or risk scores. The imbalanced dataset can be handled with PRC, which provides better insight into classifier performance, by focusing on minority classes.

The consistently high performance of MATHdist models with different thresholds highlights the robustness of the MATH approach. MATHdist <3.0 was the best model, with the highest ROC AUC, F1 score, recall, and specificity among all the MATHdist variants.

The MATH model was built upon MAT, to describe structural information better than ECFP and ST. However, MAT could be developed by augmenting information about intramolecular hydrogen bonds. Jeffrey [32] categorizes hydrogen bonds with donor and acceptor hydrogen distances (2.2–2.5Å) as “strong, mostly covalent”, (2.5–3.2Å) as “moderate, mostly electrostatic”, and (3.2–4.0Å) as “weak, electrostatic”. Most of the hydrogen bonds that exist are in the medium category. Strong hydrogen bonds require moieties or conditions that rarely occur in proteins. The average donor–acceptor distance in protein secondary structure elements is near 3.0Å [32]. Our experimental results showed that the compounds in the estrogen receptor alpha dataset showed the best results with a donor–acceptor distance with a threshold < 3.0Å, compared to a threshold below or above. It should be noted that the distance between the atoms involved does not solely determine the strength of a hydrogen bond: other factors, such as the electronegativity of the atoms, their partial charges, and the geometry of the bond, also play a significant role in determining the overall strength of the hydrogen bond.

In addition, as one of the molecular descriptors tested, the hydrogen bond parameter improves the model’s performance, because this bond influences the shape of compounds that determine the conformation in interacting with target receptors—in this case, estrogen receptor alpha. Interacting compounds as ligands with proteins as targets/receptors is analogous to “lock and key”. The conformation of the ligand must match the shape of the binding site, so that the interaction can be optimized to cause an inhibitory effect/activity. Overall, our proposed MATH models, especially MATH with distance 3.0Å, demonstrate superior performance, compared to the baseline methods.

## 5. Materials and Methods

**Figure 2:**
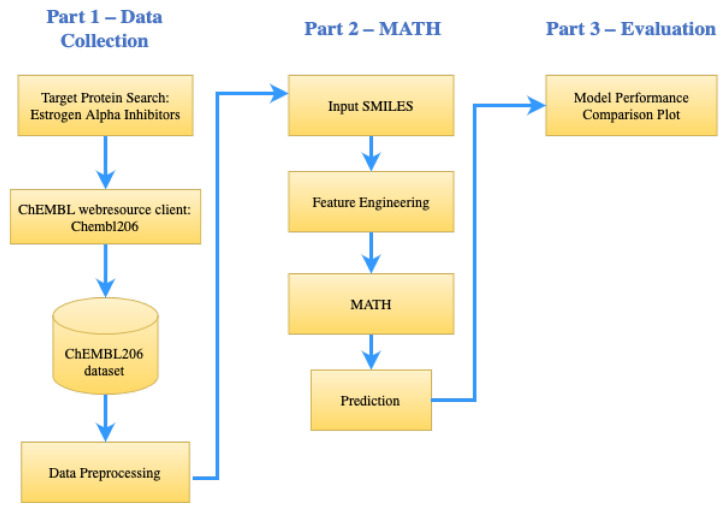
The research framework is divided into three main parts: data collection; molecular-attention Transformer plus hydrogen bond (MATH); and model evaluation. The first part explains the process of data retrieval and processing, the second part discusses the features of engineering and architecture built to make predictions, and the last part explains the evaluation of the model, along with further experiments.

The focus of this study was the classification model of QSAR, to determine the candidate of an active or inactive compound for estrogen receptor alpha inhibition in breast cancer. The model development was based on the self-attention MAT [13], by augmenting self-attention, to include additional information on the molecular description of hydrogen bonds as an intramolecular force. In this case, data addition was carried out on hydrogen bonds, to analyze whether the parameters could improve the accuracy of the molecular description task. We predicted estrogen receptor alpha inhibitors for breast cancer, by applying the molecular graph Transformer, as illustrated in Figure 2. The molecular graph Transformer was utilized to analyze and model the molecular graphs, enabling us to make accurate predictions of potential inhibitors.

### 5.1. Data Collection and Preprocessing

The bioactivity data was retrieved from the ChEMBL web source [33], specifically from Target ID ChEMBL206, which corresponds to the human estrogen receptor alpha. The ChEMBL206 dataset initially contained 5180 compounds and 46 columns of physicochemical and biological properties of molecules, as of 15 June 2023. To ensure data quality and relevance, we performed extensive preprocessing. Firstly, we removed 2109 salts, entries with missing standard values ($IC_{50}$), and known agonist compounds, resulting in a refined dataset containing 3071 compounds. Subsequently, we eliminated six duplicate canonical SMILES, leaving us with 3065 unique compounds. These preprocessing steps were crucial for ensuring the integrity and reliability of the dataset for our research.

**Figure 3:**
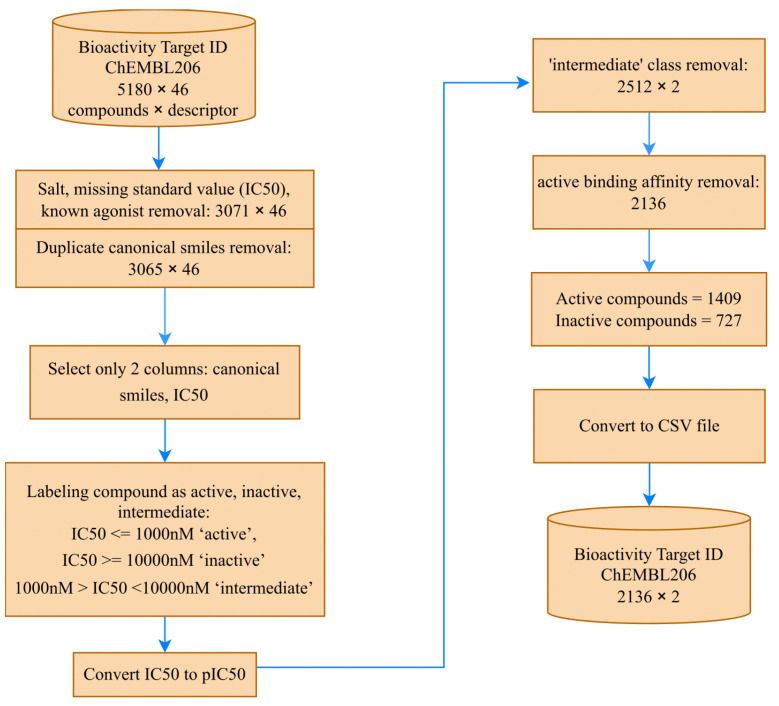
The data collection stages carried out to prepare input for the MATH feature engineering.

However, as the molecular descriptor was obtained from the input data for canonical SMILES, we decided to proceed with 3065 compounds, using only two columns—canonical SMILES and $IC_{50}$—for the subsequent steps. Figure 3 provides an overview of the data collection stage.

In the next step, the compounds were categorized as “active”, “inactive”, or “intermediate”, based on their $IC_{50}$ values: ≤1000 nM for “active”, ≥10,000 nM for “inactive”, and values in between as “intermediate” [34]. To create a binary classification prediction model for estrogen receptor alpha inhibitors, we excluded the 553 “intermediate” class compounds, leaving 2512 remaining compounds.

The ChEMBL206 dataset includes data on compound testing against estrogen receptor alpha targets as agonists, antagonists, binders, or non-binders. For our study, we focused solely on modeling the SAR of estrogen receptor alpha inhibitors or antagonists, thus excluding data related to agonist compounds. Additionally, we omitted data on active compounds that bind to estrogen receptor alpha without specific information on whether they are agonists or antagonists, as their inclusion might introduce bias to the model.

Further preprocessing involved removing known binding affinity compounds, resulting in a final dataset of 2136 compounds, comprising 1406 “active” and 727 “inactive” compounds. To achieve a more uniform distribution, the $IC_{50}$ values were converted to $pIC_{50}$ [35].

To prepare the dataset for input into the MATH model, we converted it into CSV format. Additionally, we divided the dataset into training and testing sets, to build the prediction models, with the test set comprising 20% of the compounds randomly selected from the dataset.

### 5.2. MATH

Molecule-attention Transformer plus hydrogen bond (MATH) was built upon MAT, based on Transformer architecture [15]. MATH consists of *N* multiple attention blocks, each composed of a multi-head self-attention layer and a feed-forward block with residual connections and layer normalization. A pooling layer and a classification layer follow these blocks. The multi-head self-attention layer consists of H head. Head contains *i* (i=1,…,H), which is taken as input hidden state H, and calculates:

$$\begin{array}{cc} Q_{i} & ={\mathrm{HW}}_{i}^{Q}\\ K_{i} & ={\mathrm{HW}}_{i}^{H}\\ V_{i} & ={\mathrm{HW}}_{i}^{V}. \end{array}$$

This notation is used for the attention operation in Equation (1):

(1) $$\begin{array}{c} A^{\left(i\right)}=\mathrm{softmax}\left(\frac{Q_{i}K_{i}^{T}}{\sqrt{d_{k}}}\right)V_{i}, \end{array}$$

where Q,K,V denotes a matrix with dimension $d_{k}$, which contains sets of queries, keys, and values, respectively. The dimension vector $d_{k}$ is denoted by *q* and *k*, which contain queries and keys, respectively. $W^{Q},W^{K},W^{V}$ denotes the projection matrix used to generate the different representations of queries, keys, and values.

**Figure 4:**
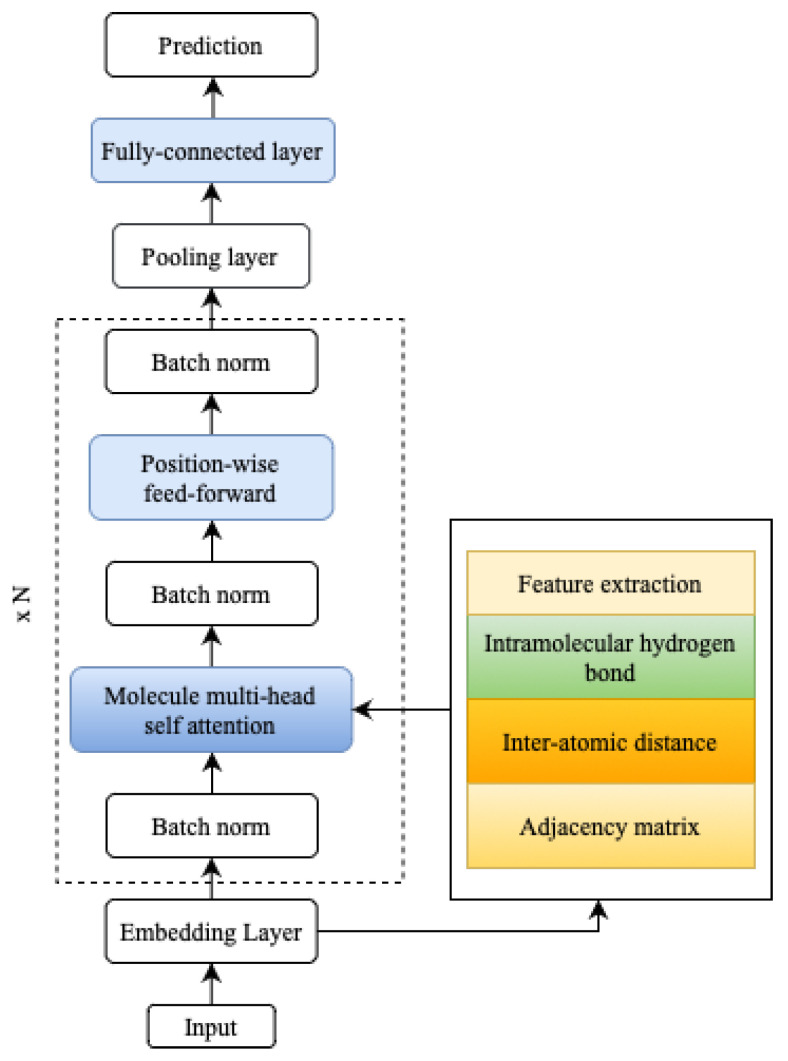
MATH architecture with additional hydrogen bond parameters.

The MATH section interprets self-attention as an adjacency matrix, a distances matrix, and hydrogen bond information between the input sequence elements, as illustrated in Figure 4. By incorporating this additional structural information, MATH moves away from using linear (textual) molecular representations as input, resulting in an improved inductive bias for the model. This inductive bias better captures the molecules’ actual structure, enhancing the model’s overall performance. An example of predicting molecular properties is usually denoted by *G* being a molecular graph with nodes representing atoms and with edges representing chemical bonds, with G=V,E being a vertex with node attribute $X_{v}$, and *E* being an edge attribute [30].

We propose a modified layer of molecule self-attention, as explained in Equation (2). The method proposed in this study is the molecule self-attention layer described in [13], with the addition of the hydrogen bond parameter, to analyze whether the hydrogen bond can reduce the error rate in predictions:

(2) $$\begin{array}{c} A^{\left(i\right)}=\left(\lambda_{a}\mathrm{softmax}\left(\frac{Q_{i}K_{i}^{T}}{\sqrt{d_{k}}}\right)+\lambda_{d}g\left(D\right)+\lambda_{g}A+\mathrm{Hbond}\right)V_{i}, \end{array}$$

where $A^{\left(i\right)}$ denotes self-attention. Let $Hbond=\{\left(i,j,d\right)|i\in HydrogenAtoms,j\in AcceptorAtoms,d\leq d_{threshold}\}$ denote the intramolecular hydrogen bond obtained from feature engineering. $A\in {\left\{0,1\right\}}^{N_{atoms}xN_{atoms}}$ denotes the graph adjacency matrix. $D\in R^{N_{atoms}xN_{atoms}}$ denotes the inter-atomic distance. $\lambda_{a},\lambda_{d},\lambda_{g}$ denote scalar values that give weights to the self-attention matrices, the inter-atomic distance, and the adjacency matrices, while *g* is the softmax for normalization.

Molecular graph descriptor extraction is the stage of acquiring the molecular graph descriptor used in the architectural model. This process takes a molecule object as input, and it generates a set of molecular graph descriptors (listed in Table 4) as the output.

**Figure 5:**
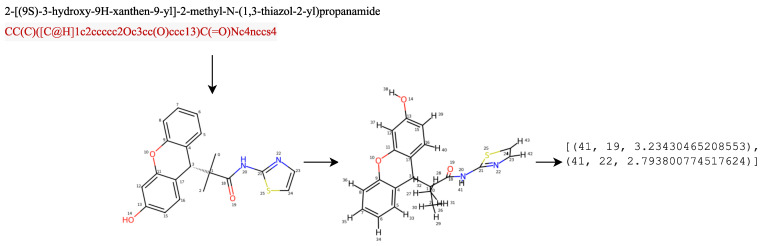
The schema intramolecular hydrogen bond of a 2-[(9S)-3-hydroxy-9H-xanthen-9-yl]-2-methyl-N-(1,3-thiazol-2-yl)propanamide compound.

**Figure 6:**
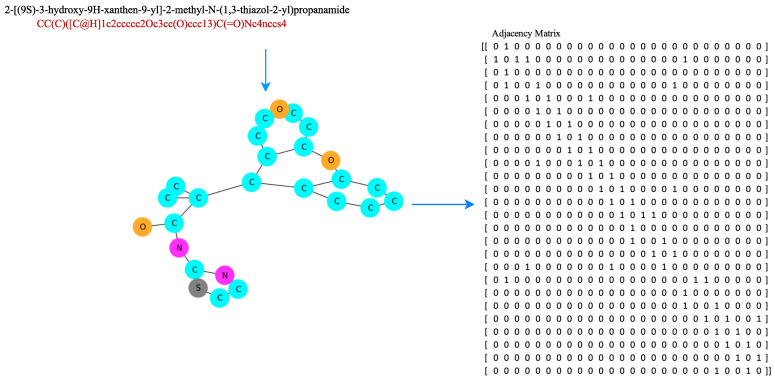
The example graph representation of a 2-[(9S)-3-hydroxy-9H-xanthen-9-yl]-2-methyl-N-(1,3-thiazol-2-yl)propanamide compound is used to obtain a molecular adjacency matrix for a downstream task.

This study focused on adding intramolecular information in the form of hydrogen bonds. In the first scenario, the hydrogen bond obtained from the feature engineering process was in the form of a matrix containing {1,0}, denoted by $H_{\mathrm{bool}}\in {\left\{0,1\right\}}^{N_{atoms}xN_{atoms}}$. The second scenario involved the addition of a hydrogen bond feature, by calculating the intramolecular hydrogen bonds within a molecule, through identifying hydrogen atoms bonded to specific acceptor atoms (nitrogen, oxygen, or sulfur) up to a certain distance threshold. It generated 3D coordinates for the molecule, iterated over the atoms, checked the necessary conditions for hydrogen bonding, and returned a list containing information about the intramolecular hydrogen bonds found in the molecule. We let $Hbond=\{\left(i,j,d\right)|i\in HydrogenAtoms,j\in AcceptorAtoms,d\leq d_{threshold}\}$, where (i,j,d) denoted a tuple containing the indices of hydrogen atoms (i) and acceptor atoms (j), together with the distance (d) between them, HydrogenAtoms was the set of indices corresponding to the hydrogen atoms in the molecule, AcceptorAtoms were the indices corresponding to a molecule’s acceptor atoms (such as nitrogen, oxygen, or sulfur), and $d_{threshold}$ was the maximum distance threshold, indicating the maximum distance allowed for hydrogen bonding considerations. Figure 5 is a scheme for obtaining hydrogen bond information from a compound, and Figure 6 is an example of a SMILES representation of a 2-[(9S)-3-hydroxy-9H-xanthen-9-yl]-2-methyl-N-(1,3-thiazol-2-yl)propanamide compound, which has 26 atoms represented as an adjacency matrix, denoted $A\in {\left\{0,1\right\}}^{N_{atoms}xN_{atoms}}$. Distance matrices were calculated from 3D conformers calculated using the RDKit package [36].

### 5.3. Model Evaluation

The MATH model is evaluated using ROC AUC, precision, recall, F1 Score, and specificity. The ROC curve is an evaluation metric commonly used for binary classification problems. This study’s classification was carried out to predict two labels: namely, active and inactive compound candidates. This curve is a probability that maps TPR (true positive rate) to FPR (false positive rate) at various threshold values, which separates ‘signal’ from ‘noise’. While AUC measures the classifier’s ability to distinguish between classes, and is used as a summary of ROC, the higher the AUC, the better the model’s performance in distinguishing between active and inactive classes. Equations (3)–(5) define the terms used in the AUC and ROC curves [37]:

(3) $$TPR=\frac{TP}{TP+FN}$$

(4) $$\begin{array}{c} \mathrm{Specificity}=\frac{TN}{TN+FP} \end{array}$$

(5) $$\begin{array}{cc} FPR & =1-\mathrm{specifity}\\ \; & =\frac{FP}{TN+FP} \end{array}$$

Meanwhile, we use a confusion matrix to calculate precision, recall, the F1 score, and specificity. These metrics are essential for assessing the effectiveness of the MATH model in predicting estrogen-receptor-alpha compounds. Precision quantifies the ratio of true positive predictions to the total positive predictions made by the model. Recall, or sensitivity, represents the ratio of true positive predictions to the actual positive instances present in the dataset. The F1 score is calculated as the harmonic mean of precision and recall, providing a balanced measure of the model’s performance, by considering both false positives and false negatives. Specificity measures the ratio of true negative predictions to the total negative instances. The precision, recall, and F1 score calculations are shown in Equations (6)–(8) [38]:

(6) $$\begin{array}{c} \mathrm{Precision}=\frac{TP}{TP+FP} \end{array}$$

(7) $$\begin{array}{c} \mathrm{Recall}=\frac{TP}{TP+FN} \end{array}$$

(8) $$\begin{array}{c} F1=2\times \frac{\mathrm{Precision}\times \mathrm{Recall}}{\mathrm{Precision}+\mathrm{Recall}} \end{array}$$

TP,FP,TN, and FN are the counts of true positive, false positive, true negative, and false negative, respectively. The MATH model is now compared to the three other candidate prediction models for estrogen-receptor-alpha compounds as the baseline.

### 5.4. Experiment Settings

The proposed approach was implemented in Python, using the Pytorch [39] package. The experiments were conducted on a Jupyter Notebook with DGX-A100 GPU. The dataset used for the experiments underwent data collection and preprocessing steps, which involved removing salts and missing standard values $\left(IC_{50}\right)$, as well as eliminating duplicate SMILES and labels for “intermediate” compounds and known agonist activity. The training set consisted of 80% of the preprocessed data, while the remaining 20% was the test set.

During the training phase, we used five-fold cross-validation, with each fold trained for 100 epochs, to obtain a robust evaluation of the model’s performance. For optimization, we employed the Adam optimizer [40] with specific hyperparameters, including an embedded atomic feature size of 1024, 8 encoder module repeats (layer number N=8), 16 molecular self-attention heads (h=16), and a batch size of 64, as suggested in Vaswani et al. [15].

To assess the performance of the proposed MATH model, we compared it to three baselines. The first baseline was MAT without intramolecular hydrogen bond augmentation [13]. The second baseline was a Transformer model by Honda et al. [18], where the SMILES textual representation was directly decoded, referred to as ST. The third baseline was an MLP model utilizing extended-connectivity fingerprinting (ECFP) [18].

Additionally, we evaluated the model’s performance under various intramolecular hydrogen bond representations. This included using a presence matrix representation for hydrogen bonds, as shown in Table 4, and considering hydrogen bonds involving different hydrogen atoms and acceptors within a distance range from 2.2 to 4.0.

## 6. Conclusions

In conclusion, our study successfully developed a deep learning model for classifying estrogen receptor alpha inhibitors, using the MATH approach with various threshold distances for hydrogen atoms and acceptors. We compared the performance of MATH to three other baseline models. The evaluation results on the testing data demonstrated that MATH with a distance of 3.0Å surpassed the performance of the other baseline models. This indicates that incorporating hydrogen bond information as one of the molecular descriptors significantly improves the classification model’s accuracy.

Hydrogen bonds play a crucial role in influencing a compound’s shape, as they determine the molecule’s conformation during its interaction with the estrogen receptor alpha. Our model provides valuable insights into compounds’ behavior and activity, by considering hydrogen bond information. Acknowledging that other molecular descriptors may further enhance the model’s accuracy is essential. Future research can explore integrating additional molecular features, to optimize the prediction model even further.

Overall, the results presented in this study hold great promise for pharmaceutical and health researchers, in guiding drug discovery pathways. The MATH approach, coupled with the consideration of hydrogen bond information, showcases a powerful tool for predicting estrogen receptor alpha inhibitors, advancing the field of drug discovery and development.

## Figures and Tables

**Table 1:** Summary of closest related work and our proposed method of molecular representation for classifying active or inactive compounds.

Study	Method	Aims/Purpose
Honda et al. [18]	SMILES Transformer (ST)	Generate SMILES Transformer (ST) fingerprints based on unlabeled SMILES data. Study molecular representations, and compare text-based models to graph convolution.
Maziarka et al. [13]	molecule-attention Transformer (MAT)	Train the MAT model with additional feature engineering, including adjacency matrix and distance matrix, into self-attention Transformer calculations and molecular graphs.
Ours	molecule-attention Transformer plus hydrogen bond (MATH)	Develop the MATH model based on MAT, with additional feature engineering of intramolecular hydrogen bonds, on self-attention Transformer calculations and molecular graphs. Feature engineering includes two representations: (1) presence of a molecule using an existence matrix of 1 and 0; (2) hydrogen bond strength (donor, acceptor, and distance).

**Table 2:** Comparison of MATH with Boolean representation to MATH using different threshold variations against multiple baselines. The first baseline is MAT without considering intramolecular hydrogen bonds by Maziarka et al. [13]. Additionally, the ST and MLP models using ECFP by Honda et al. [18] are included for reference.

	ROC AUC	Precision	Recall	F1 Score	Specificity
ST	0.770	0.571	0.707	0.631	0.559
ECFP	0.868	0.609	0.800	0.692	0.662
MAT	0.946	0.912	0.951	0.931	0.860
MATH${}_{bool}$	0.973	0.955	0.953	0.954	0.920
MATH (dist < 2.2)	0.967	0.944	0.960	0.952	0.913
MATH (dist < 2.4)	0.970	0.940	0.975	0.957	0.906
MATH (dist < 2.6)	0.971	0.943	0.958	0.950	0.910
MATH (dist < 2.8)	0.966	0.941	0.951	0.946	0.908
**MATH (dist < 3.0)**	**0.977**	**0.952**	**0.972**	**0.960**	**0.922**
MATH (dist < 3.2)	0.969	0.948	0.959	0.953	0.922
MATH (dist < 3.4)	0.962	0.933	0.977	0.955	0.892
MATH (dist < 3.6)	0.959	0.936	0.972	0.954	0.904
MATH (dist < 3.8)	0.966	0.940	0.968	0.954	0.913
MATH (dist < 4.0)	0.962	0.943	0.960	0.951	0.910

**Table 3:** Confusion matrix of the MATH (dist<3.0Å) with the highest F1 score model from five-fold cross-validation.

		Predicted Value	
		Active	Inactive
Actual label	Active	181	2
	Inactive	10	148

**Table 4:** Molecular graph descriptors generated from feature engineering.

Molecular Properties	Description
Node features	Feature vectors for each atom in the molecule.
Adjacency matrix	Matrix representing the adjacency matrix of the molecular graph. The element of the matrix is set to 1 if there is a bond between the corresponding atoms and to 0 otherwise.
Distance matrix	Matrix representing the distance matrix of the molecular graph. The element of the matrix is the pairwise distance between the atoms in the molecule.
Hydrogen Boolean (Bool)	Matrix representing the hydrogen bond matrix of the molecular graph. The element of the matrix is set to 1 if there is a hydrogen bond between corresponding atoms and to 0 otherwise.
Hydrogen bond (donor and acceptor)	Identifies and returns the indices of hydrogen atoms, acceptor atoms, and corresponding distances for intramolecular hydrogen bonds in a molecule.

## Data Availability

The data presented in this study, on human estrogen receptor alpha, are available on https://www.ebi.ac.uk/chembl/target_report_card/CHEMBL206/ (accessed on 15 June 2023).
